# Identification of a *Vibrio cholerae* chemoreceptor that senses taurine and amino acids as attractants

**DOI:** 10.1038/srep20866

**Published:** 2016-02-16

**Authors:** So-ichiro Nishiyama, Yohei Takahashi, Kentaro Yamamoto, Daisuke Suzuki, Yasuaki Itoh, Kazumasa Sumita, Yumiko Uchida, Michio Homma, Katsumi Imada, Ikuro Kawagishi

**Affiliations:** 1 Department of Frontier Bioscience, Hosei University, Kajino-cho, Koganei, Tokyo 184-8584, Japan; 2 Department of Macromolecular Science, Graduate School of Science, Osaka University, Toyonaka City 560-0043, Japan; 3 Division of Biological Science, Graduate School of Science, Nagoya University, Chikusa-ku, Nagoya, Aichi 464-8602, Japan; 4 Research Center for Micro-Nano Technology, Hosei University, Midori-cho, Koganei, Tokyo, Japan

## Abstract

*Vibrio cholerae*, the etiological agent of cholera, was found to be attracted by taurine (2-aminoethanesulfonic acid), a major constituent of human bile. Mlp37, the closest homolog of the previously identified amino acid chemoreceptor Mlp24, was found to mediate taxis to taurine as well as L-serine, L-alanine, L-arginine, and other amino acids. Methylation of Mlp37 was enhanced upon the addition of taurine and amino acids. Isothermal titration calorimetry demonstrated that a purified periplasmic fragment of Mlp37 binds directly to taurine, L-serine, L-alanine and L-arginine. Crystal structures of the periplamic domain of Mlp37 revealed that L-serine and taurine bind to the membrane-distal PAS domain in essentially in the same way. The structural information was supported by characterising the *in vivo* properties of alanine-substituted mutant forms of Mlp37. The fact that the ligand-binding domain of the L-serine complex had a small opening, which would accommodate a larger R group, accounts for the broad ligand specificity of Mlp37 and allowed us to visualise ligand binding to Mlp37 with fluorescently labelled L-serine. Taken together, we conclude that Mlp37 serves as the major chemoreceptor for taurine and various amino acids.

Bile is a biogenic detergent, by which commensal enteric bacteria and intruding pathogens are always exposed in animal intestines^1^. These bacteria have acquired resistance and/or tolerance to potentially harmful bile. *Vibrio cholerae*, the etiological agent of cholera, acquires bile resistance primarily by the expression of multiple efflux pumps^2^,^3^,^4^ and TolC^5^. Bile resistance of the bacterium also results from changes in expression of the outer membrane porins OmpU and OmpT via the transcription factor ToxR^6^,^7^,^8^. *V. cholerae* shows various responses to bile, including synthesis of virulence factors^3^,^9^, biofilm formation^10^ and motility^11^,^12^. Motility and chemotaxis of *V. cholerae* have been implicated in its pathogenicity^13^,^14^. In particular, bile and mucin from human and several other animal sources have been reported to attract *V. cholerae*^15^, suggesting that the bacteria may sense bile as a chemotactic signal to migrate toward a certain part of the intestine. This casts a sharp contrast with *Helicobacter pylori*, which inhabits the stomach and is repelled by bile^16^.

The molecular mechanisms underlying chemotaxis have been studied extensively in *Escherichia coli*^17^. Extracellular signals are sensed by methyl-accepting chemotaxis proteins (MCPs), also known as chemoreceptors. An unoccupied MCP stimulates autophosphorylation of the histidine kinase CheA, which transfers its phosphoryl group to the response regulator CheY. Phospho-CheY binds to the flagellar motor, inducing its clockwise (CW) rotation. Attractant binding to MCP inhibits CheA, resulting in counterclockwise (CCW) rotation of the flagellar motor.

The chemotaxis of *V. cholerae* involves a similar but more complicated system, a complexity that appears to contribute to its pathogenicity^13^,^14^,^18^. It has three *che* clusters which we name Che Systems I, II and III. Among them, only System II is essential for chemotaxis^19^,^20^. The functions of the other two (Systems I and III) remain unknown. *V. cholerae* also possesses many MCP-like proteins (MLPs): 44 MLPs are encoded in the genome of the classical biotype and 45 in El Tor. In a previous study, we named the *mlp* genes (from *mlp1* to *mlp45*) according to their locations in the chromosomes of the *V. cholerae* El Tor biotype^21^. Such complex chemotaxis systems have been found in various bacteria other than *E. coli*^22^. We have shown that Mlp24 (VC2161 in the El Tor biotype and VC0395_A1741 in the classical biotype), formerly called McpX^14^, is a chemoreceptor for various amino acid attractants such as l-arginine, l-proline and l-serine^21^. Deletion of the *mlp24* gene, however, does not eliminate taxis toward amino acids, suggesting that at least one additional amino acid chemoreceptor exists.

In this study, we examined bile taxis of a classical biotype strain of *V. cholerae* and found that taurine (2-aminoethylsulfonate; NH_2_CH_2_CH_2_SO_3_H) serves as a strong attractant, primarily at elevated temperature. Considering its chemical similarity to amino acids, we suspected that taurine might be sensed by some of the amino acid chemoreceptors. Indeed, we found that Mlp37, the closest homolog of Mlp24, serves as a chemoreceptor for taurine as well as serine, arginine and other amino acids. We determined the crystal structures of the ligand-binding domain of Mlp37 in complex with l-serine and taurine to reveal the molecular mechanism enabling Mlp37 to accommodate these structurally distinct ligands, l-serine and taurine. Taking advantage of the structural information, we succeeded, for the first time, in labelling a bacterial chemoreceptor, in this case Mlp37, with a fluorescent serine ligand *in vivo*.

## Results

### *V. cholerae* is attracted by taurine, a major constituent of bile

**Figure 1:**
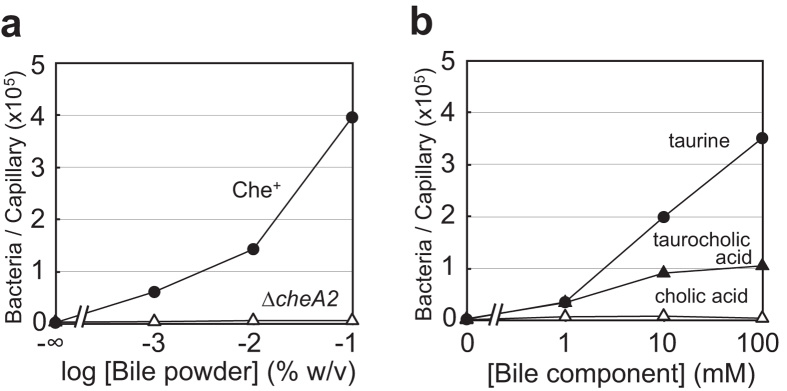
Chemotactic responses of the classical *V. cholerae* strain O395N1 to bile and bile components. (**a**) O395N1 (Che^+^, closed circles) or VcheA2 (∆*cheA2*, open triangles) cells were subjected to capillary assays with bile. (**b**) O395N1 cells were subjected to capillary assays with taurine (closed circles), cholic acid (open triangles) and taurocholic acid (closed triangles). Representative data of multiple experiments are shown.

Consistent with a previous report showing that an El Tor biotype strain exhibits chemotaxis to bile^15^, we found that the classical biotype strain O395N1 in the O1 serogroup, showed an attractant response to bile (Fig. 1a). In contrast, the *E. coli* strain RP437 and the *Vibrio parahaemolyticus* strain LM4070 did not show any significant response to bile (Supplementary Fig. S1a,b). We examined which ingredient(s) of bile act as attractants, testing cholic acid, deoxycholic acid, taurine, taurocholic acid and taurodeoxycholic acid. Glycine, another component of bile, was excluded as it was already known to attract *V. cholerae*^21^ and *E. coli*^23^. Taurine elicited a strong attractant response from *V. cholerae* (Fig. 1b), but it did not attract *E. coli*, *V. parahaemolyticus*, or *Vibrio alginolyticus* (Supplementary Fig. S1g–i). It should be noted that the latter three species showed robust attractant responses to l-serine (Supplementary Fig. S1d–f). Taurocholic acid and taurodeoxycholic acid also served as weak attractants, whereas cholic acid and deoxycholic acid did not (Fig. 1b and Supplementary Fig. S1c). These results suggest that taurine is a key constituent of bile that attracts *V. cholerae*.

### Identification of a chemoreceptor involved in taurine taxis of *V. cholerae*

**Figure 2:**
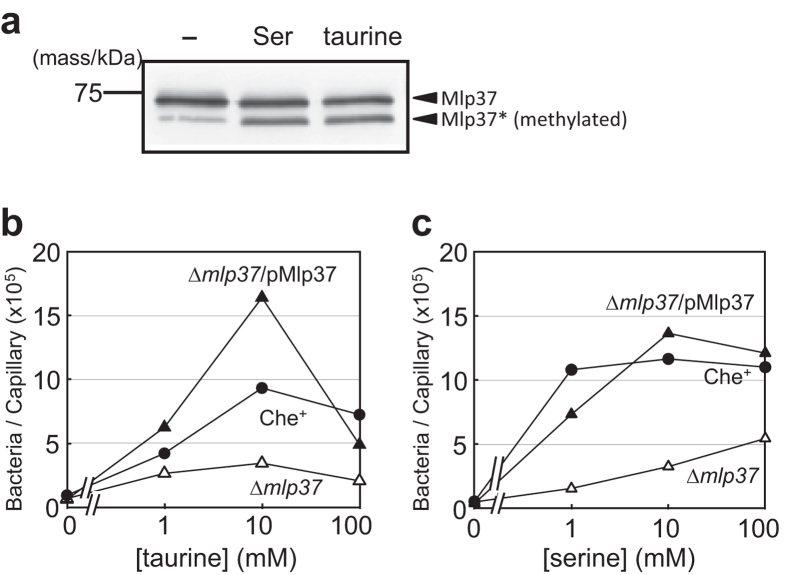
Mlp37 mediates chemotaxis responses to taurine and amino acids. (**a**) Effects of taurine and serine on methylation of Mlp37. Wild-type (O395N1) cells expressing Mlp37-FLAG were incubated with no chemoattractant (−), 10 mM serine (Ser) or taurine (taurine) for 30 min at 30 °C. Mlp37-FLAG was detected by immunoblotting with anti-FLAG antibody. (**b**,**c**) Effects of the *mlp37* deletion on chemotactic responses to taurine (**b**) and serine (**c**). O395N1 (Che^+^) cells carrying the vector pAH901 (O395N1/pAH901, closed circles), Vmlp37 (∆*mlp37*) cells carrying the vector (O395N1-37/pAH901, open triangles) or the Mlp37-FLAG-expressing plasmid pMlp37 (O395N1-37/pMlp37, closed triangles) were subjected to capillary assays with taurine or all the individual proteiogenic amino acids (summarised in Supplementary Table S1). Representative data of multiple experiments are shown.

The chemical nature of taurine (2-aminoethanesulfonate) and the broad ligand specificity of the amino acid chemoreceptor Mlp24^21^ prompted us to examine whether that receptor can also sense taurine. We first examined methylation of Mlp24 (67.7 kDa) upon addition of taurine. Methylation of an MCP increases its mobility upon SDS-PAGE, which can be detected by immunoblotting^24^. Unlike l-serine, a high concentration (10 mM) of taurine enhanced methylation only slightly (Supplementary Fig. S2d)^21^. Furthermore, all the other functional and biochemical assays we carried out showed that the contribution of Mlp24 to taurine taxis, if any, is limited (Supplementary Fig. S2a–c). We therefore turned our attention to Mlp37, the closest homolog of Mlp24. The *mlp37* gene (VCA0923 in El Tor/VC0935_0316 in classical) encodes a polypeptide with a calculated molecular mass of 70.3 kDa. Mlp37 has 49% identity and 63% similarity to Mlp24 in amino acid sequence and like Mlp24, contains a CACHE domain^25^ and a pre-CACHE motif^21^. We found that the N-terminal extension of 28 amino acid residues is missing in Mlp37 orthologs in other *Vibrio* species (Supplementary Fig. S3). We therefore deemed this extension dispensable for function and cloned the ‘truncated’ coding region of Mlp37 (residues 29 to 652; 67.1 kDa) into the vector pAH901^21^. A sequence encoding the FLAG tag was fused to the sequence encoding the C-terminus of Mlp37 to yield plasmid pMlp37. The *V. cholerae* strain O395N1 was transformed with pMlp37. Resulting transformants were tested for methylation of the Mlp37-FLAG protein. Mlp37 became methylated in the presence of taurine, l-serine (Fig. 2a), glycine, l-alanine, l-cysteine or l-arginine, whereas l-glutamate, one of the weakest attractants, had no effect (Supplementary Fig. S4a,b). These results suggest that Mlp37 mediates attractant responses to taurine, l-serine and a number of other amino acids. We therefore hypothesised that Mlp37 plays a major role in taurine taxis.

### *mlp37* is required for taxis to taurine and amino acids

To confirm the role of Mlp37 in chemotaxis, we constructed the *mlp37*-deleted strain Vmlp37 (O395N1 ∆*mlp37*). In the capillary assay for chemotaxis, the mutant strain showed greatly reduced responses toward taurine (Fig. 2b). Its tactic responses to each of the twenty proteinogenic amino acids are shown in Table S1. Chemotactic responses of the ∆*mlp37* mutant to l-serine, l-alanine, l-cysteine, l-arginine, l-asparagine, l-threonine, l-lysine and l-valine and glycine were markedly decreased. Most of these defects were complemented by expressing Mlp37 in *trans* from plasmid pMlp37 (Fig. 2c and Supplementary Table S1). These results, together with the methylation results (Fig. 2a), strongly argue for the involvement of Mlp37 in attractant responses to taurine and various amino acids.

### Binding of taurine and amino acids to the periplasmic fragment of Mlp37

**Figure 3:**
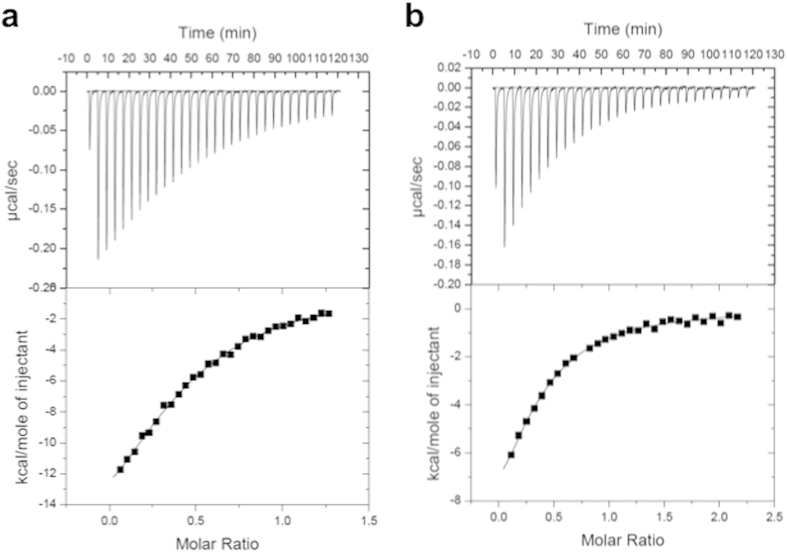
Binding of taurine and serine to the periplasmic fragment of Mlp37 (Mlp37p). ITC measurements with10 μM Mlp37p were carried out with 0.2 mM taurine (attractant, **a**) or serine (attractant, **b**). Enthalpy changes per mol were plotted as a function of the molar ratio of L-serine to Mlp37p.

To examine whether attractants bind directly to the periplasmic domain of Mlp37, we employed isothermal titration calorimetry (ITC) with a periplasmic fragment of Mlp37 (residues 58–303; named Mlp37p). The titration curves of purified Mlp37p (10 μM) with taurine (Fig. 3a) show direct binding with a dissociation constant (*K*_d_) of 3.2 μM, calculated by assuming that an Mlp37p monomer binds one taurine molecule. ITC analyses also showed that l-serine, l-alanine and l-arginine bind directly to Mlp37p with *K*_d_ values of 3.6 μM, 2.7 μM and 5.6 μM, respectively (Fig. 3b, Supplementary Fig. S4c,d). In contrast, l-glutamate, which was not sensed by Mlp37 in the capillary assay, did not show appreciable binding to Mlp37p (Supplementary Fig. S4e). The affinity of Mlp37p for l-arginine is equivalent to that of Mlp24p, and the affinities for l-serine and l-alanine are one order of magnitude higher than those for Mlp24p^21^. These results demonstrate that Mlp37 is a chemoreceptor for taurine, l-serine, l-alanine and l-arginine.

### Three-dimensional structures of Mlp37p in complex with taurine

**Figure 4:**
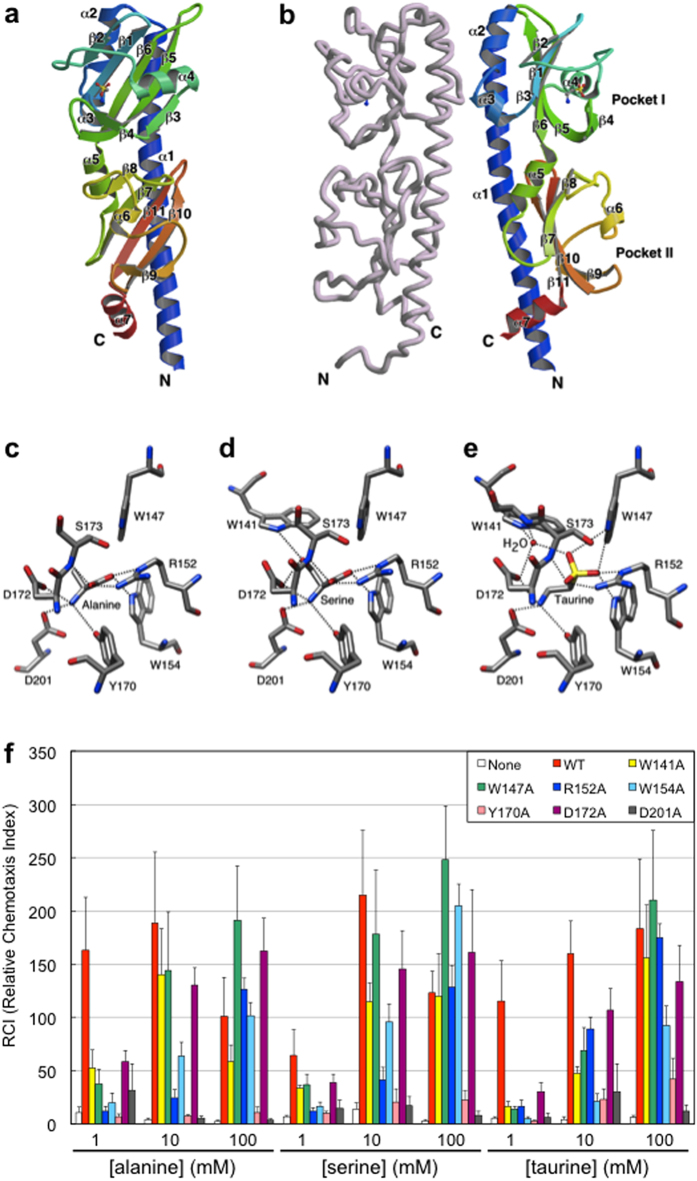
Structural basis of ligand recognition by Mlp37. Structures of the Mlp37p-ligand complexes (**a–e**). (**a**) Ribbon drawing of the Mlp37p-taurine complex monomer. The model is colour-coded from blue to red from the N- to the C-terminus, with labels showing the secondary structure elements. The bound taurine is shown as a ball-and-stick model. (**b**) The Mlp37p-taurine complex dimer. One subunit is shown in the same colour as (**a**), and the other is pale pink. It should be noted that the ribbon monomer image is rotated 90° from the one in A. (**c–e**) Close up view of the ligand-binding sites of the l-alanine complex (PDB ID: 3C8C) (**c**), the serine complex (**d**), and the taurine complex (**e**). Possible hydrogen bonds are indicated by dotted lines. (**f**) Effects of alanine substitutions of residues in pocket I on attractant responses. Capillary assays were carried out for Vmlp201 (∆*mlp24* ∆*mlp37*) cells expressing wild-type or mutant Mlp37: none, open bars; wild-type, red; W141A, yellow; W147A, green; R152A, blue; W154A, cyan; Y170A, pink; D172A, purple; D201A, grey bars. Capillaries were filled with TMN buffer containing the indicated concentrations (1, 10 or 100 mM) of each attractant.

The periplasmic domain of Mlp37 (VCA0923) of an El Tor strain (PDB ID: 3C8C) forms a homodimer, each subunit of which consists of two tandemly linked PAS-like domains^26^. This structure belongs to a family of bacterial sensor domains^27^,^28^. It provides two potential ligand-binding pockets per monomer (the membrane distal and proximal ones are hereafter referred to as pockets I and II, respectively). The 3C8C structure was solved in a complex with l-alanine, in which alanine was bound to pocket I. Among the known ligands for Mlp37, only taurine is not an l-amino acid. Although the structure of taurine is quite distinct from that of amino acids, its dissociation constant is comparable to those for l-serine and l-alanine. To elucidate the ligand recognition mechanism of Mlp37, we solved the crystal structures of Mlp37p bound to taurine and l-serine. The overall structures of the taurine and l-serine complexes are almost the same as that of the l-alanine complex (PDB ID, 3C8C) (Supplementary Fig. S5a,b). However, the space group and cell dimensions of these crystals differ from one another (Supplementary Fig. S5c–e) (Supplementary Table S2). The taurine complex, like the l-alanine complex, forms a dimer with a neighbouring molecule in the asymmetric unit. In contrast, the l-serine complex forms a dimer with a molecule related by crystallographic two-fold symmetry (Supplementary Fig. S5c–e). In spite of these differences in molecular packing, the dimer structures are basically identical (Supplementary Fig. S5a–e). α2 and the C-terminal region of α1 stabilise the dimer by forming an up-and-down four-helix bundle structure with its dimer mate (Fig. 4a,b).

Taurine and l-serine are bound only in pocket I in the crystal structures. The amino acid backbone atoms of bound l-serine are located at essentially the same positions as those of the bound l-alanine molecule in the 3C8C structure (Fig. 4c,d). The amino group of l-serine is in contact with Tyr-170, Asp-201 and Asp-172, which are triangularly arranged around it. The carboxyl group of l-serine interacts mainly with the guanidino group of Arg-152. The indole NH of Trp-154 and the main-chain amino group of Ser-173 also contribute to the binding of the carboxyl group of the l-serine ligand. These interactions are exactly conserved in the l-alanine complex (Fig. 4c,d). The side-chain hydroxyl group of l-serine is recognised by Asp-172 and the indole NH of Trp-141 (Fig. 4d).

The binding of taurine is remarkably similar to that of l-serine (Fig. 4d,e and Supplementary Fig. S5f). The amino group of taurine lies at the equivalent position of that of l-serine and interacts with Tyr-170, Asp-201 and Asp-172. Two oxygen atoms of the sulfonate group are located in the corresponding positions to the carboxyl oxygen atoms of l-serine, but they are out from the plane formed by the guanidino group of Arg-152. Thus, the indole NH of Trp-147 seems to contribute to the interaction in conjunction with the guanidino group of Arg-152, the indole NH of Trp-154 and the main-chain amino group of Ser-173. The remaining oxygen of the sulfonate is located near the hydroxyl oxygen of l-serine, and it interacts with the indole NH of Trp-147 and a water molecule bound to Asp-172, the indole NH of Trp-141, and the main-chain amino group of Ala-174. These structural features are essentially the same in the two subunits of the dimeric Mlp37p taurine complex (Supplementary Fig. S5g). The residues in contact with taurine can be superimposed onto the corresponding residues of the l-serine complex (Supplementary Fig. S5f), with root mean square deviations of about 0.3 Å (0.314 for A-subunit and 0.265 for B-subunit), indicating that taurine mimics l-serine in Mlp37.

The entrance of Pocket I of one subunit in the l-alanine bound dimer is in a fully closed conformation that embraces the ligand (Supplementary Fig. S6). In contrast, that of the other subunit has an opening almost identical to those in l-serine and the taurine complexes. The distance between the indole nitrogen of Trp-141 and one of the carboxyl oxygen atoms of Asp-172 in the closed conformation is 3.2 Å, indicating that Trp-141 in the loop connecting β2 and α4 interacts directly with Asp-172 in the loop connecting β4 and β5. In contrast, these residues in the l-serine and taurine complexes are much further apart, because Trp-141 and Asp-172 interact with the hydroxyl group of the l-serine ligand and with a water molecule in the taurine complex. As a result, the entrances to pocket I in the l-serine and taurine complexes are slightly open. Such an opening would allow larger amino acid R groups of other amino acids to stick out from the pocket without significantly affecting interactions with the amino acid backbone atoms, a situation that could explain the ability of Mlp37 to sense a diversity of amino acids. The opening, however, is not wide enough to allow passage of an attractant molecule itself. Thus, ligand binding must involve some type of open-to-closed conformational change in pocket I.

### Mutational analysis of attractant recognition

To examine the *in vivo* roles of the residues in contact with the ligands (Trp-141, Trp-147, Arg-152, Trp-154, Tyr-170 Asp-172 and Asp-201), we constructed mutant receptors with individual alanine replacements at each residue. Ser-173 was not tested because ligand interaction involves its main chain. The mutant receptors were expressed in strain Vmlp201, which lacks *mlp24* and *mlp37* and therefore shows only residual tactic responses to amino acids. Although the expression levels of all of the mutant proteins were essentially the same as that of the wild-type protein (Supplementary Fig. S4e), all showed defects in mediating taxis toward l-alanine, l-serine and taurine (Fig. 4f). The most profound defects were observed at the lowest attractant concentration tested (1 mM in the capillary) (Fig. 4f). Cells expressing the Y170A and D201A proteins showed impaired chemotaxis even at higher attractant concentrations (10 or 100 mM in the capillary), suggesting that recognition of the amino group by these residues is essential for ligand binding. It should be noted that cells respond to attractants diffusing out from the mouth of the capillary; therefore, the attractant concentration the cells encounter when migrating up the gradient are much lower than those in the capillary. Cells expressing other mutant proteins showed nearly wild-type performance at high attractant concentrations (10 or 100 mM in the capillary), implying that these mutants have reduced affinities for the attractants. Each mutation had similar effects on responses to the three attractants tested, as was expected from the similarities in the crystal structures. These results reinforce the importance of these residues in recognition of the attractants, l-alanine, l-serine and taurine.

The crystal structures suggest that pocket II is not involved in attractant sensing, as was shown for Mlp24^21^ and *Bacillus subtilis* chemoreceptors^29^,^30^. To confirm this hypothesis, we substituted alanine for two of the well-conserved potential ligand-binding residues in pocket II, Y221A and H234A that are well conserved among the related amino acid chemoreceptors of different species. In contrast to the pocket I mutations, the pocket II mutations had mild to negligible effects (Supplementary Fig. S7a,b), demonstrating that recognition of taurine and amino acids involves pocket I, but not pocket II.

### Observation of Mlp37 with a fluorescently labelled ligand

**Figure 5:**
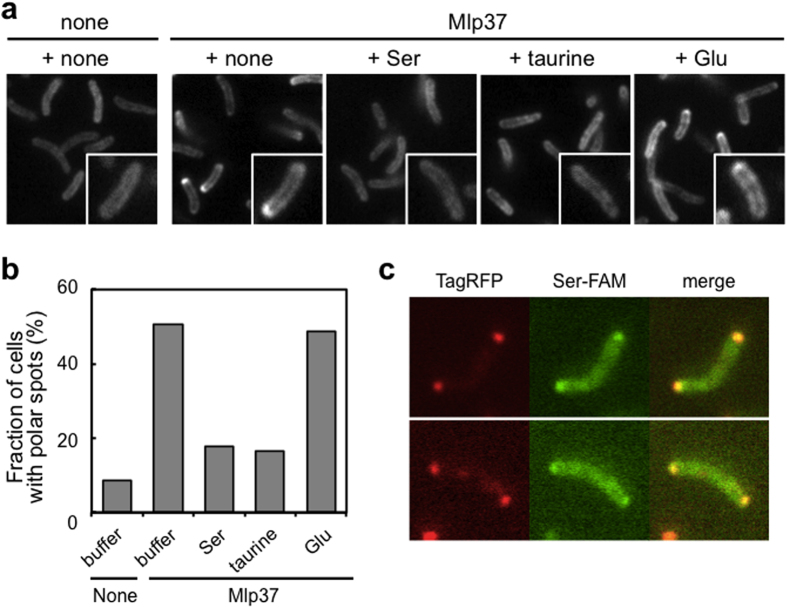
Imaging of Mlp37 with a fluorescently labelled ligand. (**a**) Competitive labelling of Mlp37 with carboxyfluorescein-labelled l-serine [Ser-FAM]. Vmlp201 (∆*mlp24* ∆*mlp37*) cells carrying the vector (none) or pMlp37 (Mlp37) were incubated in TMN buffer containing 100 μM Ser-FAM supplemented with none (+none), 1 mM serine (+Ser), 1 mM taurine (+taurine), or 1 mM glutamate (+Glu). (**b**) The fraction of cells with polar Ser-FAM spots in the absence or presence of an attractant. (**c**) Co-localisation of Ser-FAM with Mlp37. Vmlp201 cells expressing Mlp37-TagRFP were treated with Ser-FAM: left, Mlp37-TagRFP; middle, Ser-FAM; right, merge.

The small openings in the pocket I binding sites for serine and taurine prompted us to test whether localisation of Mlp37 can be visualised *in vivo* with a fluorescently labelled amino acid: l-serine 5(6)-carboxyfluorescein ester (Supplementary Fig. S7d, hereafter referred to as Ser-FAM), in which carboxyfluorescein is linked to the hydroxyl group of l-serine through an ester bond. When treated with Ser-FAM, fluorescent spots were observed at the poles of ∆*mlp24* ∆*mlp37* (Vmlp201) cells expressing Mlp37 from pMlp37, but not for cells carrying the empty vector (Fig. 5a,b). Pre-incubation with a 10-fold molar excess of l-serine or taurine before Ser-FAM labelling dramatically decreased the occurrence of polar fluorescent spots. By contrast, pre-incubation with l-glutamate, one of the weakest attractants, did not affect Ser-FAM labelling (Fig. 5a,b). These results suggest that l-serine and taurine compete with Ser-FAM for binding to Mlp37. To confirm that the polar fluorescent spots correspond to Ser-FAM bound to Mlp37, we constructed a plasmid encoding Mlp37-TagRFP (named pKRB116). This fluorescent fusion protein expressed in strain Vmlp201 (∆*mlp24* ∆*mlp37*) supported essentially wild-type chemotaxis toward serine (Supplementary Fig. S7b) and showed polar localisation (Fig. 5c). When treated with Ser-FAM, cells expressing Mlp37-TagRFP (Vmlp201/pKRB116) showed Ser-FAM spots that coincided with Mlp37-TagRFP (Fig. 5c). These results not only confirm the mechanism of ligand recognition by Mlp37 but also provide, for the first time, a tool to visualise ligand binding to a bacterial chemoreceptor *in vivo*.

## Discussion

This study demonstrated that the classical biotype of *V. cholerae* shows attractant chemotaxis to taurine. This finding is consistent with the previous report that the El Tor biotype of *V. cholerae* is attracted by bile^15^, as taurine is a major component of bile. Taurine taxis can also account for the previous observation that bile enhances the motility of *V. cholerae* in semisolid agar^12^.

Several lines of evidence support the conclusion that the MCP-like protein Mlp37 mediates taxis to taurine and various amino acids. First, the deletion of *mlp37* severely affected taxis to taurine and several amino acids and those defects were complemented by a plasmid expressing the *mlp37* gene. Second, methylation of Mlp37 was enhanced in the presence of taurine and various amino acids. Third, ITC measurements with a periplasmic fragment of Mlp37 (Mlp37p) demonstrated direct binding of Mlp37p to taurine as well as several amino acids.

The crystal structures of Mlp37p in a complex with attractant ligands argue strongly that pocket I, the membrane-distal of the tandem PAS-like domains in Mlp37p, undergoes a substantial (open-to-closed-type) conformational change upon ligand binding. The l-serine complex, in which the hydroxyl group of the ligand points toward the small opening of pocket I, reveals the molecular basis for the diversity of amino acids that can be sensed by Mlp37. An enlarged opening could accommodate the longer R groups of l-arginine, l-lysine and l-glutamine. Remarkably, taurine and l-serine binding involve the same set of Mlp37 residues with almost identical three-dimensional arrangements. These observations readily explain why the dissociation constant of taurine is close to that of l-serine. The taurine complex structure also suggests why taurocholic acid and taurodeoxycholic acid act as weak attractants, whereas cholic acid and deoxycholic acid do not. The amino group of taurine lies near the entrance of the binding pocket, and the entrance is slightly open (Supplementary Fig. S6). In taurocholic acid and taurodeoxycholic acid the acidic groups are linked to the amino group of taurine. If the pocket entrance opens a little more, it could accommodate the tauroconjugates by binding the taurine moiety in the pocket while leaving the cholic and deoxycholic moieties outside of the pocket. However, the binding affinity may be greatly reduced because some of the interactions with the amino group of taurine would be lost in this configuration.

The slightly open conformation of pocket I in the serine complex enabled us to visualise ligand binding *in vivo* with a fluorescently labelled amino acid. Binding of Ser-FAM to Mlp37, which can be outcompeted by excess amounts of serine or taurine, validates the structural information. It also provides a novel tool to study the cell biology of bacterial chemotaxis.

The ligand complex structures and the mutational analyses demonstrate that only one of the two potential ligand-binding pockets (the membrane-distal pocket I) is involved in ligand binding by Mlp37. This observation is consistent with previous reports on the related amino acid chemoreceptors, McpB of *B. subtilis*^29^ and Mlp24 of *V. cholerae*^21^. Some histidine kinases with two tandem PAS-like domains have also been reported to bind their ligands through pocket I^31^,^32^. There has been no evidence that pocket II in any MCP or histidine kinase with tandem PAS-like domains binds small ligands except that the membrane-proximal PAS-like domain of McpC interacts with lipid-anchored ligand-binding proteins^30^.

What is the physiological relevance of taurine taxis? Taurine is abundant in the intestines of humans and other vertebrates, including fish. Marine invertebrates have high concentrations of taurine that serves as an osmolyte that regulates tissue osmolarity^33^. *Vibrio* species are very often associated with fish and aquatic invertebrates^34^. Chemoattraction to taurine could therefore enhance fitness of *V. cholerae* when it is associated with various hosts, particularly marine organisms. The fact that cultivation of the wild-type strain at 37 °C enhanced taurine chemotaxis dramatically (and l-serine chemotaxis to a lesser extent) (Supplementary Fig. S1j,k), however, argues strongly that taurine chemotaxis is advantageous primarily in the intestines of a mammalian host rather than in marine/estuarine animals. Because bile is excreted from the gallbladder to the small intestine, taxis to taurine could also play a role in pathogenicity, as has been shown for chemotaxis to amino acids^21^. It is possibe that a taurine gradient allows *V. cholerae* to remain in the small intestine, which is their preferred site of colonisation in humans, for a longer time. It has been argued that bile serves as a repellent of *V. cholerae* and that this behaviour would help the bacteria to escape from the lumen by penetrating through the protective glycocalyx of the mucous that overlays the epithelium^3^,^9^. Chemotaxis toward higher concentrations of taurine would be disadvantageous in this respect. However, the mucus layer of the small intestine contains various amino acids^15^, which should attract *V. cholerae* cells and facilitate their migration through the mucosa toward the epithelium. Upon penetration into the mucus layer or adherence to epithelial cells, an unidentified sensor could detect a set of amino acids (asparagine, arginine, glutamate and serine) to turn on the *ctxAB* operon that encodes cholera toxin, as has been discussed previously^21^,^35^.

Our finding that *V. cholerae* can respond to taurine as well as various amino acids raises the intriguing possibility that the pathogen senses other host factors to migrate toward favourable environments as it passes through the gastrointestinal tract. Indeed, *V. cholerae* has more than forty MLPs, most of them without known functions. Comprehensive studies on the chemotaxis behaviour of *V. cholerae* within the host would extend our understanding of the mechanisms that underlie infection by, and the virulence of, this serious world-wide pathogen.

## Methods

### Bacterial strains

The classical biotype strain O395N1 is wild type for chemotaxis (Che^+^) and its derivative VcheA2 lacks *cheA2*^19^ is defective in general chemotaxis (Che^—^). Strains Vmlp24 (∆*mlp24*) and Vmlp201 (∆*mlp24* ∆*mlp37*) are derivatives of O395N1 lacking *mlp24* and/or *mlp37*^21^. Vmlp37 (∆*mlp37*) was constructed similarly in this study. The *E. coli* strain RP437^36^ is Che^+^. The *V. parahaemolyticus* strain LM1017^37^ and the *V. alginolyticus* strain YM4^38^ are wild type for polar flagellation and chemotaxis but defective in lateral flagellation (Pof^+^ Laf^−^ Che^+^). The *E. coli* strain HCB436^39^ lacks the methylesterase CheB and the methyltransferase CheR as well as the chemoreceptors.

### Construction of plasmids

Plasmid pMlp37 was constructed as follows. From genomic DNA of strain O395N1, the region encoding Mlp37 missing the N-terminal extension (see Results for details) was amplified by PCR to introduce restriction sites (EcoRI and SphI) at the 5′ and 3′ ends, respectively. The resulting DNA fragment was digested with these restriction enzymes and cloned into similarly digested pAH901^21^, yielding pMlp37, which encodes the FLAG-tagged Mlp37. Site-directed mutagenesis of *mlp37* was carried out with QuickChange II Site-directed Mutagenesis Kit (Agilent Technologies/Stratagene, CA), using pMlp37 as a template.

The DNA fragment encoding the entire periplasmic domain (residues 58–303) of Mlp37 was amplified and subcloned into the expression vector pGEX-6P-2 so that the Mlp37-encoding sequence was fused in-frame to the 3′ of the GST-encoding sequence to yield pGEX-Mlp37p.

For construction of a plasmid encoding Mlp37-TagRFP, the TagRFP coding region was amplified by standard PCR using plasmid pTagRFP-C (Evrogen) as a template with primers designed for cloning and introducing a 3× Gly linker at the N-terminus of TagRFP. The amplified fragment was cloned into plasmid pTWV228 (Takara Bio) to yield pKRB112 expressing free TagRFP with the linker. The *mlp37* gene was amplified using pMlp37 as a template and was cloned into plasmid pKRB112 to yield pKRB116 expressing the Mlp37-TagRFP fusion. Expression of the resulting proteins was verified by immunoblot using anti-TagRFP antibody (Evrogen).

All the cloned and mutated genes were verified by DNA sequencing.

### Capillary assay

Chemotactic ability was examined by a capillary assay as described previously^21^. In brief, overnight culture of *V. cholerae* cells grown in TG medium (1% tryptone, 0.5% NaCl, 0.5% glycerol) at 30 °C was diluted 1:30 into fresh TG medium, shaken at 30 °C for 6 h, harvested and washed with TM buffer (50 mM Tris-HCl [pH 7.4], 5 mM glucose, 5 mM MgCl_2_). Cells were resuspended in TMN buffer (50 mM Tris-HCl [pH 7.4], 5 mM glucose, 100 mM NaCl, 5 mM MgCl_2_) (to O.D._590_ = 0.1). After pre-incubation of cells at 30 °C for 1 h, a capillary containing an amino acid solution was inserted into the cell suspension and incubated for another 1 h. The number of bacteria in the capillary was estimated by plating serial dilutions on LB agar.

### Methylation assay

Receptor methylation was examined by immunoblotting with an anti-FLAG M2 antibody (Sigma) as described previously^21^.

### Expression and purification of the periplasmic fragment of Mlp37p

Strain BL21(DE3) (Novagen) carrying pGEX-Mlp37p was cultured in LB broth Lennox (Nacalai Tesque, Inc., Kyoto Japan) containing 50 μg/ml of ampicillin at 37 °C until the cell density had reached an OD_600_ of about 0.8. Isopropyl β-d-1-thiogalactopyranoside (IPTG) was then added to a final concentration of 0.1 mM to induce protein expression, and the culture was continued for 12 hours at 20 °C. Cells were harvested by centrifugation, suspended in phosphate buffered saline (PBS) (137 mM of NaCl, 2.7 mM of KCl, 10 mM of Na_2_HPO_4_•12H_2_O, and 1.8 mM of KH_2_PO_4_ at pH 7.4) and disrupted by sonication. After removing cell debris by centrifugation, the cell lysate was loaded to a Glutathione-Sepharose^TM^ 4B column (GE Healthcare) followed by washing with PBS. Proteins were eluted with 50 mM Tris-HCl (pH 8.0) buffer containing 10 mM reduced glutathione. The N-terminal GST tag was then cleaved using PreScission^TM^ Protease (GE Healthcare), and the resulting sample was dialysed in 1.0 L dialysis buffer (50 mM Tris-HCl pH 7.0, 150 mM NaCl, 1 mM DTT, and 1 mM EDTA) at 4 °C for 12 hours using Spectra/por Dialysis Membrane MWCO 6,000–8,000 (Spectrum Laboratories, Inc.). The protein solution was loaded again to a Glutathione-Sepharose^TM^ 4B column (GE Healthcare) to remove GST and unreacted protein, and further purified by gel filtration chromatography with a High Load 26/60 Superdex 200 gel filtration column in 20 mM Tris-HCl and 150 mM NaCl (pH 8.0). The peak fraction was collected and concentrated.

### Isothermal titration calorimetry (ITC)

Purification of GST-fused Mlp24p and Mlp37p (encoded in plasmid pGEX-Mlp24p and pGEX-Mlp37p) and titrations of the periplasmic fragments with ligands using a VP-ITC micro-calorimeter (MicroCal Inc., Northampton, MA) were carried out essentially as described previously^21^,^40^.

### Statistical analysis

Triplicate data were analysed by statistical hypothesis testing (Kolmogorov-Smirnov test, *F*-test and *t*-test) using an application programme “R” [R Development Core Team (2012). R: A language and environment for statistical computing. R Foundation for Statistical Computing, Vienna, Austria. ISBN 3-900051-07-0, URL http://www.R-project.org/].

### Crystallisation, X-ray data collection and structure determination of the Mlp37p complexes with taurine and serine

Crystallisation of the Mlp37p complexes with l-serine and taurine was performed by the hanging-drop vapour-diffusion technique. Crystallisation drops were prepared by mixing Mlp37p solution containing 10 mM l-serine or 10 mM taurine with an equal volume of a reservoir solution. Initial screening was carried out using the following screening kit: Wizard I and II, Cryo I and II (Emerald Biostructures) and Crystal Screen I and II (Hampton Research), and then the conditions were optimised. Crystals appeared within 3 days. The final crystallisation conditions are summarised in Table S2. The taurine complex crystal belongs to an orthorhombic space group of *P*2_1_2_1_2_1_ with unit cell dimensions of *a* = 33.2 Å, *b* = 119.1 Å and *c* = 138.8 Å. The l-serine complex crystallised in an orthorhombic space group of *C*222_1_ with unit cell dimensions of *a* = 98.7 Å, *b* = 134.2 Å and *c* = 50.5 Å. The l-serine complex was soaked in a solution containing 90% (v/v) of the reservoir solution and 10% (v/v) MPD for a few seconds before transfer into liquid nitrogen. X-ray diffraction data were collected at 95 K under nitrogen gas flow at the synchrotron beamline BL41XU of SPring-8 (Harima, Japan), with the approval of the Japan Synchrotron Radiation Research Institute (JASRI) (Proposal No. 2012B1462 and 2013A1406). The data were processed with MOSFLM^41^ and scaled with SCALA^42^. Initial phase was calculated by molecular-replacement using the Phenix programme suite^43^ with the Mlp37p-alanine complex structure (PDB ID: 3C8C) as a search model. The atomic model was constructed with Coot^44^ and refined with Phenix to 1.8 Å for the l-serine complex and to 1.95 Å for the taurine complex. During the refinement process, iterative manual modifications were performed. The final refinement R factor and the free R factor of the l-serine complex were 18.7% and 21.4%, respectively. The Ramachandran plot indicated that 95.9% and 4.1% residues were located in the most favourable and allowed regions, respectively. The refinement R factor and the free R factor of the taurine complex were converged to 19.0% and 23.7%, respectively. The Ramachandran plot indicated that 92.9% and 7.1% residues were located in the most favourable and allowed regions, respectively. Data collection and refinement statistics are summarised in Supplementary Table S2.

### Fluorescence microscopy

Cells were grown at 30 °C in tryptone glycerol (TG) broth [1% tryptone, 0.5% NaCl, 0.5% (w/v) glycerol] supplemented with 50 μg/ml ampicillin. Overnight cultures were diluted 50-fold into fresh TG medium and incubated for 4 h at 37 °C. Cells were harvested by centrifugation at room temperature and resuspended in 100 μl of TMN buffer [50 mM Tris-HCl (pH-7.4), 5 mM glucose, 100 mM NaCl] and 100 μM Ser-FAM [l-serine 5(6)-carboxyfluorescein ester] (Eurofins Genomics Inc., Tokyo) was added. If necessary, non-labelled l-serine or taurine (final concentration: 1 mM) was added to TMN buffer. Cells were then incubated for 30 min at 37 °C, and washed once with TMN buffer. An aliquot of the cell suspension was spotted onto a MAS-coated glass slide (Matsunami Glass Inc., Osaka). Cells were then observed under a fluorescence microscope (Olympus IX71) equipped with a 100× oil-immersion objective lens. GFP and TagRFP were visualised using fluorescence mirror units, U-MNIBA3 and U-MWIG3 (Olympus), respectively. All images were recorded and processed by using a cooled charge-coupled-device camera ORCA-ERII (Hamamatsu Photonics) and the software Metamorph ver. 7.6 (Universal Imaging).

## Additional Information

**Accession codes:** The atomic coordinates have been deposited in Protein Data Bank, www.pdb.org (PDB ID code 5AVE and 5AVF).

**How to cite this article**: Nishiyama, S.-i. *et al.* Identification of a *Vibrio cholerae* chemoreceptor that senses taurine and amino acids as attractants. *Sci. Rep.* **6**, 20866; doi: 10.1038/srep20866 (2016).

## Supplementary Material

Supplementary Information
