# Supplementary material for: Identification of a Vibrio cholerae chemoreceptor that senses taurine and amino acids as attractants
Source: Sci Rep. 2016 Feb 16;6:20866. doi: 10.1038/srep20866 (PMC4754685; doi:10.1038/srep20866)
Supplement: Supplementary Information [file srep20866-s1.pdf]

Supplementary Information

Identification of a *Vibrio cholerae* chemoreceptor that senses taurine and amino acids as attractants

So-ichiro Nishiyama, Yohei Takahashi, Kentaro Yamamoto, Daisuke Suzuki, Yasuaki Itoh,
Kazumasa Sumita, Yumiko Uchida, Michio Homma, Katsumi Imada, and Ikuro Kawagishi

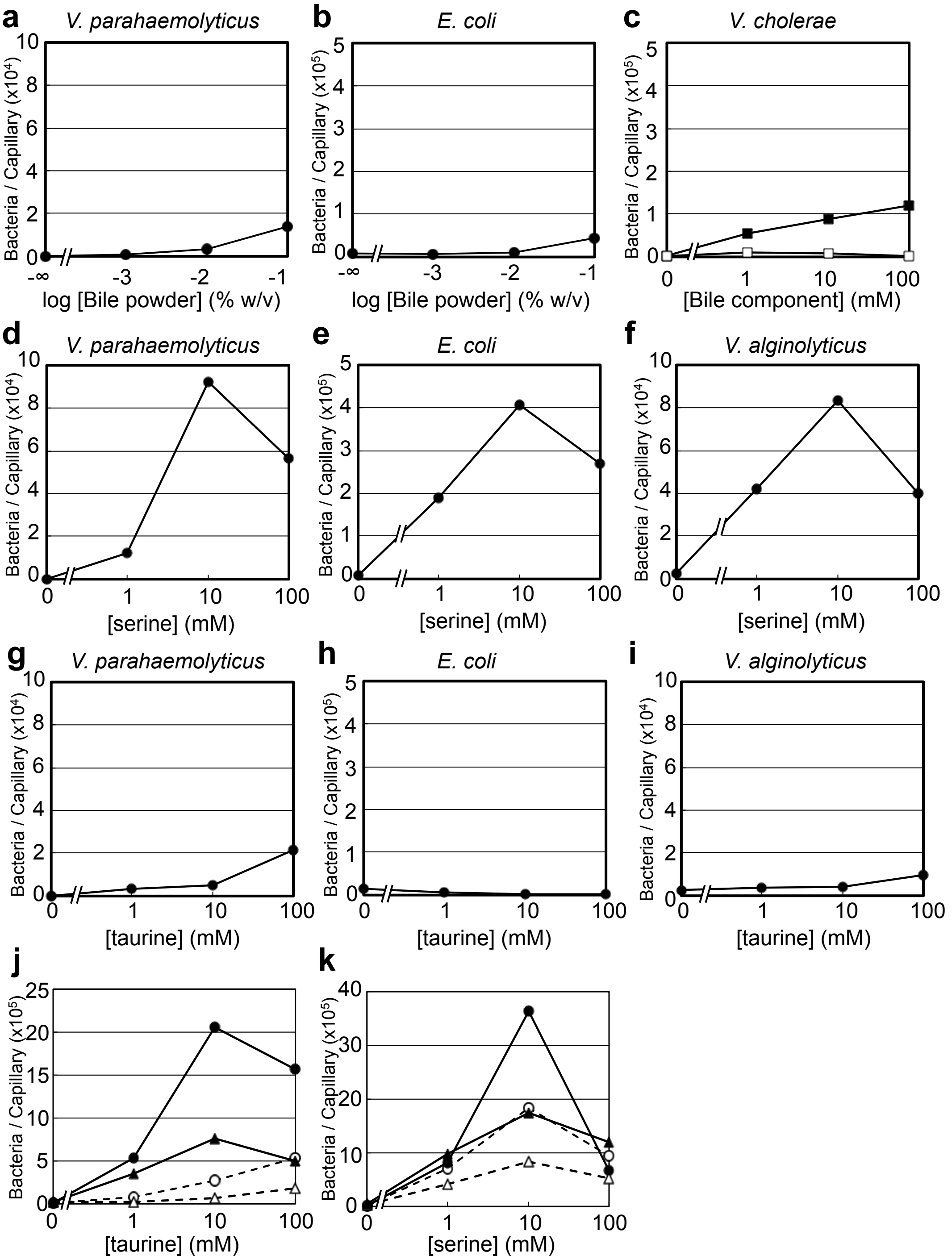

Figure S1 | Chemotactic responses of various bacterial species to bile, bile components, and serine. (**a, b**) Responses of *V. parahaemolyticus* strain LM4070 (**a**) and *E. coli* strain RP437 (**b**) to bile (**c**). Responses of *V. cholerae* O395N1 to deoxycholic acid (open squares) and taurodeoxycholic acid (closed squares). (**d-i**) Responses of *V. parahaemolyticus* (**d, g**), *E. coli* (**e, h**) and *V. alginolyticus* strain YM4 (**f, i**) to serine (**d-f**) and taurine (**g-i**). (**j, k**) Effects of culture temperature on taurine and serine taxis. Wild-type (circles) or Δ*mlp37* (triangles) cells cultured at 30ºC (open symbols, dotted lines) or 37ºC (closed symbols, solid lines) were subjected to capillary assays at 30ºC with taurine (**j**) or serine (**k**).

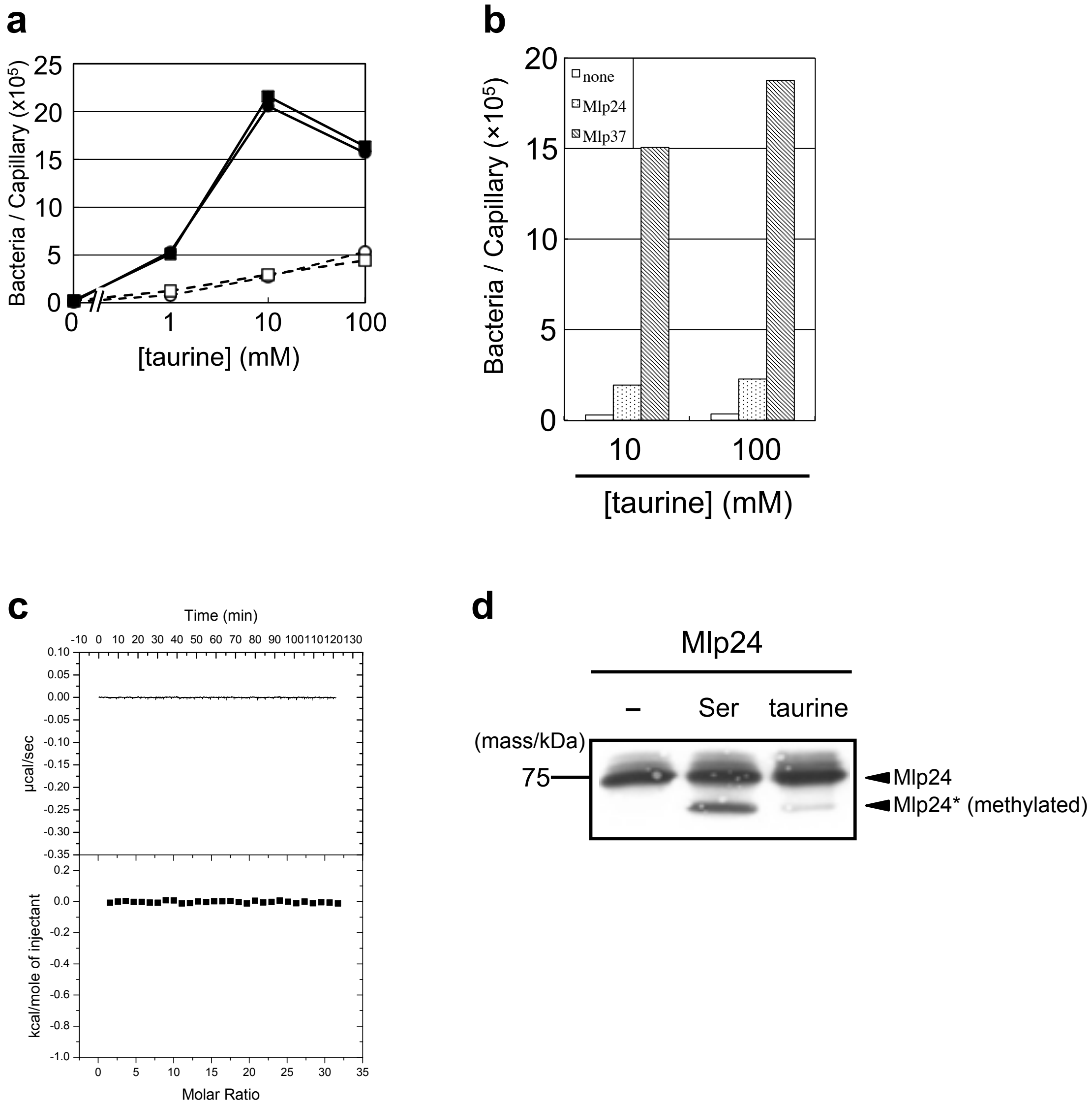

Figure S2 | Examination of taurine-sensing ability of Mlp24. (**a**) Effects of culture temperature on taurine taxis of the wild-type (circles) and Δ*mlp24* (squares) strains. Cells cultured at 30ºC (open symbols, dotted lines) or 37ºC (closed symbols, solid lines) were subjected to capillary assays at 30ºC with taurine. Data of wild-type cells are the same as those shown in Fig. 5b. (**b**) Taurine responses of Vmlp37 (Δ*mlp37*) cells carrying the vector (pAH901, open bars) or the plasmid encoding Mlp24-FLAG (pMlp24, dotted bars) or Mlp37-FLAG (pMlp37, hatched bars). Capillary assay was carried out as described in Material and Methods. (**c**) ITC analysis of 10 µM Mlp24p with 5 mM of taurine. Enthalpy changes per mol were plotted as a function of the molar ratio of taurine to the periplasmic fragment of Mlp24 (Mlp24p). The binding parameters calculated from the data are described in the text. (**d**) Methylation patterns of Mlp24 in the presence and absence of taurine or L-serine. Vmlp201 cells expressing Mlp24-FLAG were incubated with no chemoattractant (−), 10 mM taurine, or 10 mM L-serine (Ser) at 30ºC for 30 min and subjected to immunoblotting as described in Material and Methods.

a

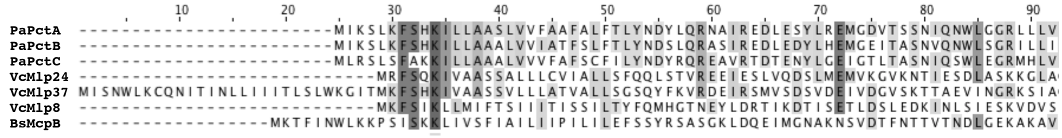

b

```
5'-CTATAAGCGCGCAGTTGCACAAAATTTCAATATAAATCAAATTATTATCACAACCTCCGAATAAAAGT
                                                                 No SD?

   GTGATCTCTAACTGGCTGAAGTGCCAAAACATAACTATAAATTTATTAATAATAATAACCCTATCGTT
   ------------------------------------->
   (1)

   ATGGAAAGGAATCACG ATG AAA TTC AGC CAT AAA ATT GTT ...-3'
         SD?        ------------------------------------>
                    fMet Lys Phe Ser His Lys Ile Val ...
                    (29)
```

Figure S3 | The N-terminal extension of Mlp37. (**a**) Alignment of the amino acid sequences of Mlp37 and related MLPs. The *mlp37* ORF annotated in the database (GenBank #: CP000626.1) has an unorthodox extension sequence (28 aa) at the N terminus. (**b**) Nucleotide sequence upstream of the second start codon of the "short" *mlp37* ORF. The deduced Shine-Dalgarno sequence is indicated with a box. The "short" Mlp37 was demonstrated to be fully functional as described in the text.

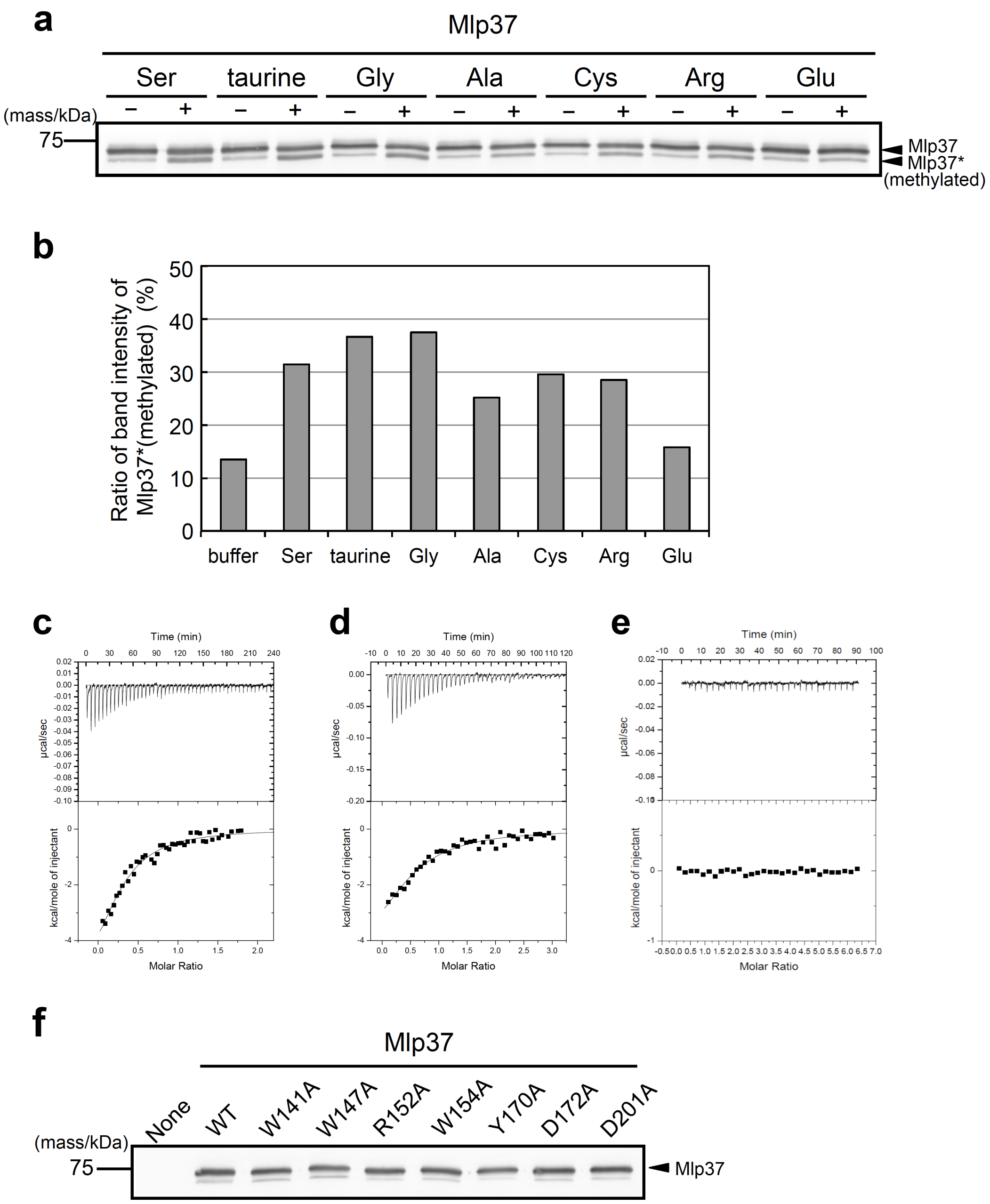

Figure S4 | Ligand specificity of Mlp37. (**a**) Methylation patterns of Mlp37 in the presence of L-serine (Ser), taurine (taurine), glycine (Gly), L-alanine (Ala), L-cysteine (Cys), L-arginine (Arg) or L-glutamate (Glu). O395N1 cells (classical) expressing Mlp37-FLAG were incubated with (+) or without (−) each amino acid (10 mM) at 30ºC for 30 min and the resulting cells were subjected to immunoblotting as described in Materials and Methods. Samples equivalent of 20 µl each of cell suspensions (OD_{600} = 1.0) were applied. **(b)** Quantification of the methylation levels. For each sample, intensities of Mlp37 bands were measured using ImageJ (http://imagej.nih.gov/ij/) and the ratios of the methylated band (*i.e.* lower bands in Fig. S4a) are shown. (**c-e**) Binding of L-alanine (**c**), L-arginine (**d**), and L-glutamate (one of the weakest attractant, **e**) to the periplasmic fragment of Mlp37 (Mlp37p). ITC measurements of 10 µM Mlp37p were carried out with 0.1 mM of L-alanine (**c**), 0.2 mM of L-arginine (**d**) or 1 mM of L-glutamate (**e**). Enthalpy changes per mol were plotted as a function of the molar ratio of serine to Mlp37p. The binding parameters calculated from the data are described in the text. (**f**) Cellular amounts of the mutant Mlp37 proteins used for the capillary assays (Fig. 4f). Whole cell extracts of Vmlp201 (Δ*mlp24* Δ*mlp37*) cells carrying the vector plasmid pAH901 (none) or the plasmid encoding the wild-type (WT) or mutant pMlp37 protein (W141A, W147A, R152A, W154A, Y170A, D172A, or D201A) were subjected to immunoblotting as described in Methods. Samples equivalent of 5 µl each of cell suspensions (OD_{600} = 1.0) were applied.

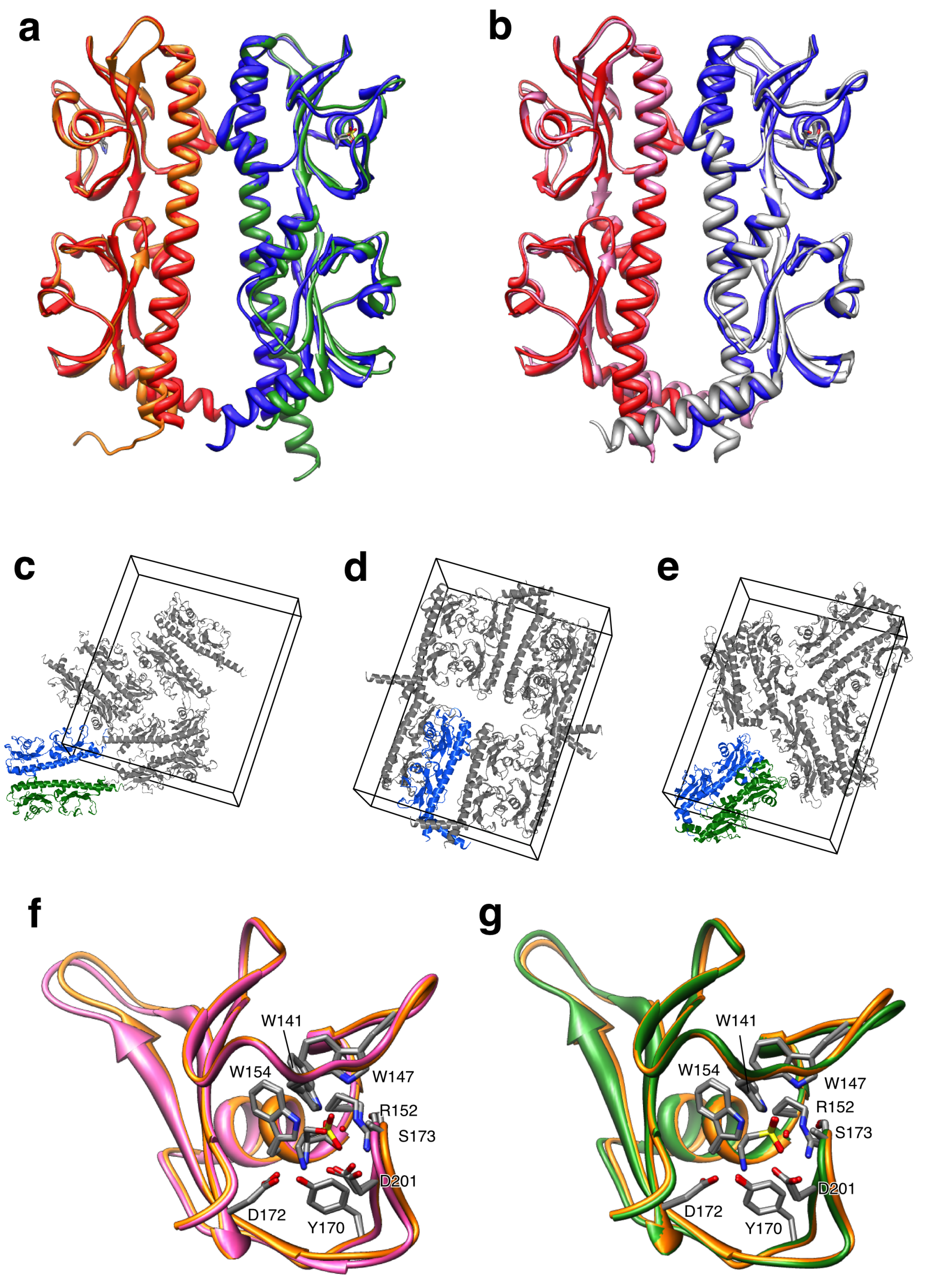

Figure S5 | Comparison of the overall and pocket I structures of the Mlp37p complexes with different ligands. (**a-e**) Comparison of the overall structures of the Mlp37p complexes. (**a**) The taurine complex dimer (orange and forest green) is superimposed onto the L-alanine complex dimer (red and blue). (**b**) The L-serine complex dimer (pink and light grey) is superimposed onto the L-alanine complex dimer (red and blue). (**c-e**) Differences in molecular packing between the taurine complex (**c**), the L-serine complex (**d**), and the L-alanine complex (PDB ID: 3C8C) (**e**). The molecules in an asymmetric unit is coloured in blue and green, and other symmetry related molecules are in grey. The unit cells are shown with black line. (**f-g**) Structural comparison of pocket I in the Mlp37p-ligand complexes. (**f**) Pocket I of the L-serine complex (pink) is superimposed onto that of the taurine complex subunit a (orange). The residues involved in the ligand recognition are shown in stick model. (**g**) The pocket I structures of the taurine complex subunit a (orange) and b (green) are superimposed. No significant structural difference is observed in the two subunits.

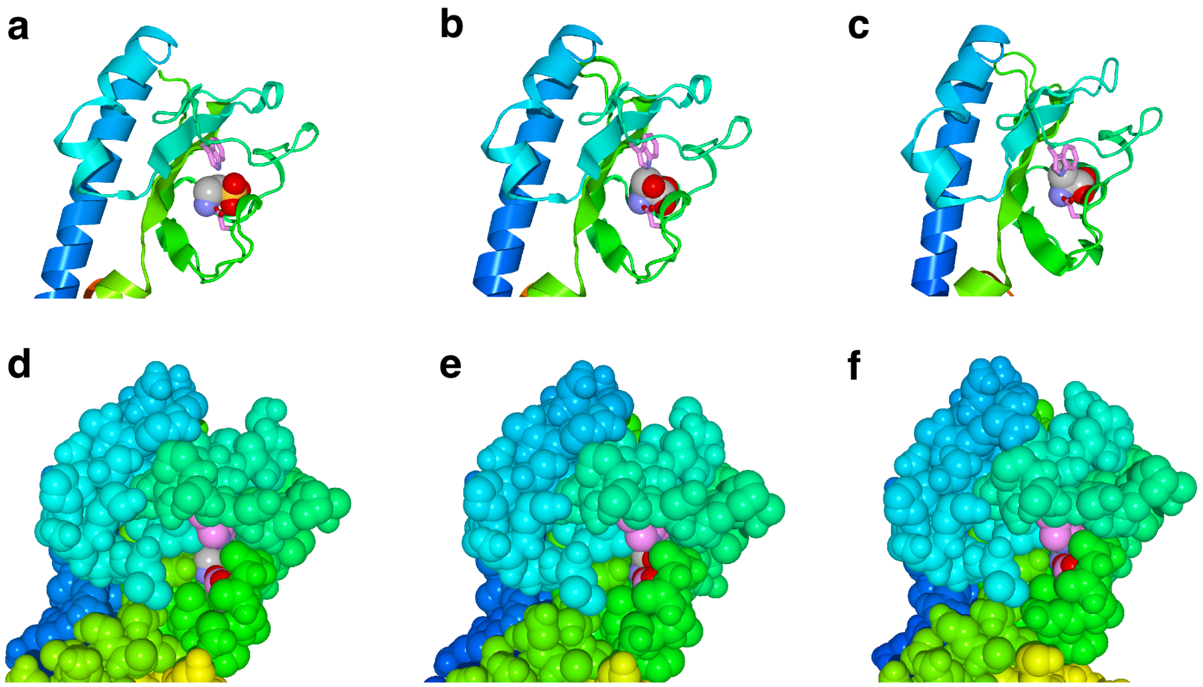

Figure S6 | Entrance of the ligand binding pocket. (**a, b, c**) The pocket I structures of the taurine (**a**), the L-serine (**b**) and the L-alanine (**c**) complexes are drawn in ribbon representation. The bound taurine, L-serine and L-alanine molecules are shown as ball models, and Trp-141 and Asp-172 are as stick models. (**d, e, f**) The pocket I structures of the taurine (**d**), the L-serine (**e**) and the L-alanine complex (**f**) are shown as ball models painted with the same colour as (**a**), (**b**) and (**c**). The pocket entrances of the taurine and the L-serine complexes are slightly open and the ligand atoms can be seen (**a, b, d, e**), whereas that of the L-alanine complex is fully closed (**c, f**).

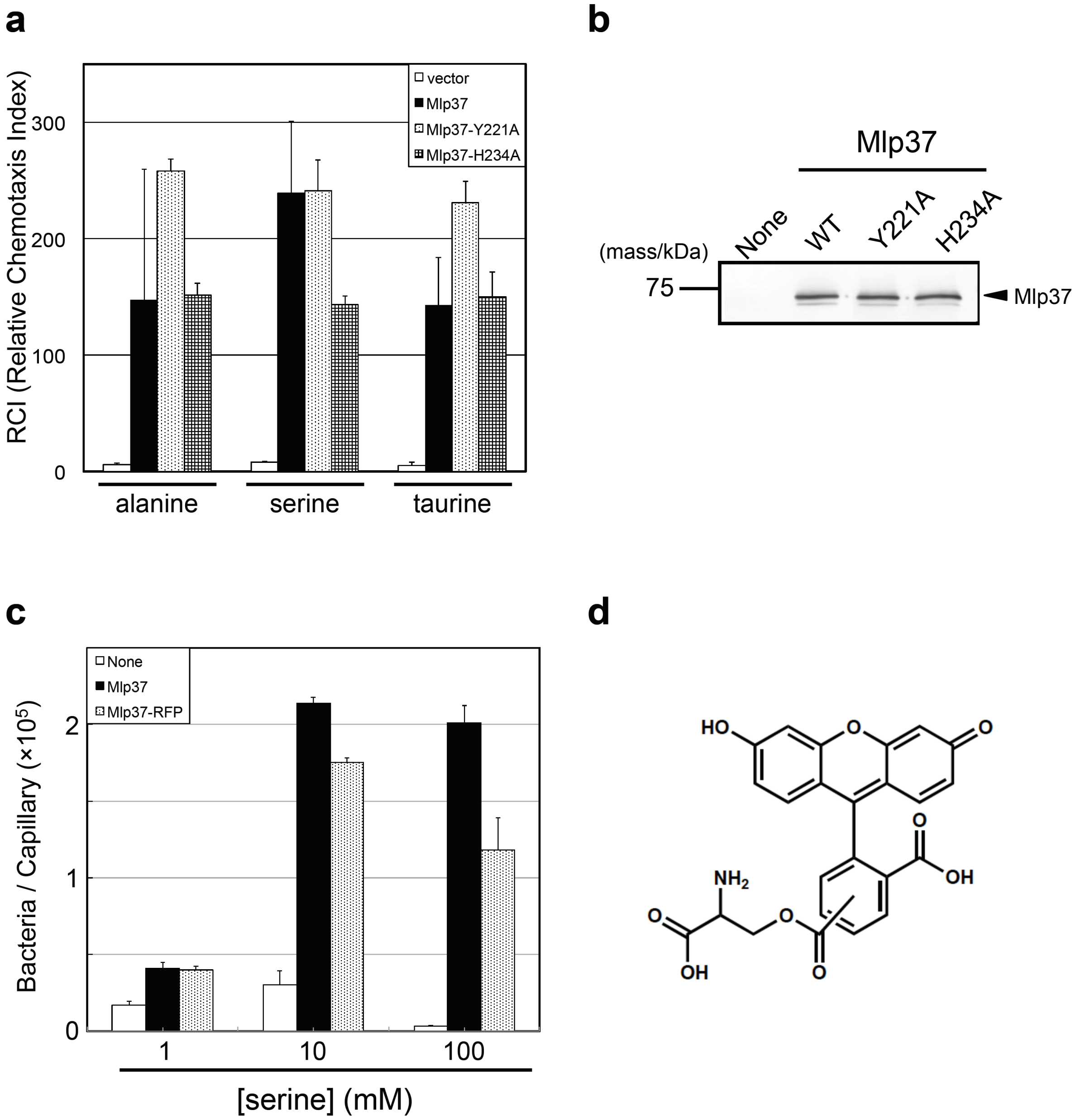

Figure S7 | Effects of alanine substitutions in pocket II and the RFP tag on attractant responses. (**a**) Capillary assays were carried out for Vmlp201 (Δ*mlp24* Δ*mlp37*) cells expressing wild-type or mutant Mlp37: none, open bars; wild-type, closed bars; Y221A, dotted bars; or H234A, cross-hatched bars. Capillaries were filled with TMN buffers containing 10 mM each attractant. (**b**) Cellular amounts of the mutant Mlp37 proteins used for the capillary assay (Fig. S4a). Whole cell extracts of Vmlp201 (Δ*mlp24* Δ*mlp37*) cells carrying the vector plasmid pAH901 (None) or the plasmid encoding the wild-type (WT) or mutant pMlp37 protein (Y221A and H234A) were subjected to immunoblotting as described in Methods. Samples equivalent of 5 μl each of cell suspensions (OD_{600} = 1.0) were applied. (**c**) Capillary assays were carried out for Vmlp201cells carrying the vector (pAH901, open bars) or the plasmid encoding Mlp37-FLAG (pMlp37, closed bars) or Mlp37-TagRFP (pKRB116, dotted bars). Capillaries were filled with TMN buffer containing 1, 10 or 100 mM serine. (**d**) The structural formula of Ser-FAM.

Table S1 | Chemotactic responses of classical *V. cholerae* strains: O395N1 carrying pAH901 (Che$^+$), Vmlp37 carrying pAH901 (Δ*mlp37*) and Vmlp37 carrying pMlp37 (Δ*mlp37*/pMlp37).

L-amino acid	Number of cells in the capillary (x 10^3) [a]		
	classical		
	Che$^+$ [b]	Δ*mlp37* [c]	Δ*mlp37*/ pMlp37 [d]
serine	855±174 [e]	*336±91	**887±62
glycine	586±74	**234±25	**702±21
alanine	541±42	**192±39	**663±63
cysteine	522±97	**57±19	399±214
arginine	504±45	**171±37	**679±6
asparagine	427±60	*200±79	214±66
histidine	370±96	224±98	479±181
threonine	344±24	*260±35	**473±57
lysine	328±63	*184±45	**380±36
glutamine	323±40	227±63	**394±4
proline	322±5	149±119	334±74
methionine	288±84	276±72	488±116
valine	253±17	*165±42	**533±27
phenylalanine	154±47	*60±29	**246±51
glutamic acid	137±37	73±62	78±21
asparatic acid	120±35	105±70	97±26
leucine	111±23	130±23	**248±8
tryptophan	103±19	94±8	112±67
isoleucine	101±22	*70±3	108±28
tyrosine	68±22	46±40	67±36

[a]Number of cells intruded into a capillary containing 10 mM of each amino acids were evaluated as described in materials and methods.

[b]The values for the wild-type strain (Che$^+$) are the same as those shown in the previous study (21).

[c]For each amino acid, the value was tested in comparison with that of "Che$^+$" and is marked if it is significantly lower at the level of $P < 0.05$ (*) or $P < 0.01$ (**) in the *t*-test.

[d]For each amino acid, the value was tested in comparison with that of "Δ*mlp37*" and is marked if it is significantly higher at the level of $P < 0.05$ (*) or $P < 0.01$ (**) in the *t*-test.

[e]The mean and the standard deviations (SD) of three independent experiments are shown.

Table S2 | Summary of crystallization, X-ray data collection, and refinement statistics

	Mlp37p-taurine	Mlp37p-serine
**Crystallization**		
Protein conc. (mg/ml)	9.82	13.8
Drop vol. (μL) (protein, reservoir solution)	1.0, 1.0	1.0, 1.0
Reservoir solution		
Volume (mL)	1.0	1.0
Buffer	0.1 M MES pH 6.5	0.1M Phosphate-citrate pH 4.2
Precipitant	15% (*w/v*) PEG-8000	10% (*w/v*) PEG-8000
Additive	30% 2-methyL-2,4-pentanediol	0.2M NaCl
Temperature (K)	293	293
**Data collection**		
Space group	$P2_12_12_1$	$C222_1$
Cell dimensions, a, b, c (Å)	33.2, 119.1, 138.8	98.7, 134.2, 50.5
Wavelength (Å)	1.0000	1.0000
Resolution (Å)	33.3-1.95 (2.06-1.95)	32.0-1.8 (1.9-1.8)
R_{merge}	9.6 (37.1)	9.7 (41.1)
I/σI	13.7 (5.2)	11.4 (4.7)
Completeness (%)	99.9 (99.8)	99.5 (99.2)
Redundancy	7.2 (7.0)	7.2 (7.3)
**X-ray refinement statistics**		
Resolution (Å)	32.3-1.95 (2.00-1.95)	31.7-1.8 (1.86-1.8)
Number of reflections		
Working set	40,573 (2,584)	31,367 (2,650)
Test set	2,000 (134)	1,583 (160)
R_w (%)	19.3 (22.3)	18.8 (26.4)
R_{free} (%)	23.6 (31.6)	21.4 (31.6)
Rms deviation		
Bond length (Å)	0.008	0.006
Bond angle (°)	1.03	1.01
B-factors		
Protein atoms	36.2	26.1
Ligand atoms	36.3	26.6
Solvent atoms	38.4	32.0
Ramachandran plot (%)		
Most favored	92.9	95.9
Additionally allowed	6.9	4.1
Generously allowed	0.2	0
Disallowed	0	0
Number of atoms		
Protein	3,775	1,972
Ligand	14	7
Solvent	397	207

Values in parentheses are for the highest resolution shell.

$R_w = \Sigma \| Fo | - | Fc \| / \Sigma | Fo |$, $R_{free} = \Sigma \| Fo | - | Fc \| / \Sigma | Fo |$
